# Determinants of contraceptive continuation among women in sub-Saharan Africa

**DOI:** 10.1186/s12905-023-02578-8

**Published:** 2023-08-24

**Authors:** Eugene Budu, Joshua Okyere, Mary Dansoah Osei, Abdul-Aziz Seidu, Bright Opoku Ahinkorah

**Affiliations:** 1 Korle Bu Teaching Hospital, P. O. Box, 77, Accra, Ghana; 2 Department of Population and Health, University of Cape Coast, Cape Coast, Ghana; 3 Department of Nursing, College of Health Sciences, Kwame Nkrumah University of Science and Technology, Kumasi, Ghana; 4 Centre For Gender and Advocacy, Takoradi Technical University, P.O.Box 256, Takoradi, Ghana; 5 College of Public Health, Medical and Veterinary Sciences, James Cook University, Townsville, QLD 4811 Australia; 6 School of Public Health, Faculty of Health, University of Technology Sydney, Sydney, Australia

**Keywords:** Contraceptive continuation, Women, Demographic and health survey

## Abstract

**Background:**

Contraceptive continuation is an important factor that has significant implications on total fertility rates and reproductive health outcomes, like unintended pregnancies. Therefore, it is imperative to understand the factors that influence women’s decision to continue the use of contraceptives. The present study examined the determinants of contraceptive continuation among women in sub-Saharan Africa (SSA).

**Methods:**

Data for the study were extracted from the most recent Demographic and Health Surveys (DHS) of twenty-four (24) countries in SSA. Descriptive and multivariable binary logistic regression analysis were conducted. Frequencies, percentanges, and an adjusted odds ratio with 95% confidence intervals were used to present the results.

**Results:**

Compared to adolescents, adult women aged 45–49 years [aOR: 1.24; CI: 1.13–1.37] had higher odds of contraceptive continuation. The odds of contraceptive continuation were lower among those working [aOR: 0.96; CI: 0.93–0.98] compared to those not working. Also, the study shows that the likelihood of contraceptive continuation was lower among those exposed to family planning messages compared to those not exposed [aOR: 0.91; CI: 0.88–0.93]. Compared to women who used LARCs, women who used pills [aOR: 0.34; CI: 0.33–0.36], injectable [aOR: 0.42; CI: 0.40–0.43], other modern contraceptives [aOR: 0.72; CI: 0.68–0.75] or traditional methods [aOR: 0.50; CI: 0.478–0.523] were less likely to continue with their contraception. Women with one birth [aOR: 0.86; CI: 0.83–0.90] and those with 2 + births in the last five years [aOR: 0.54; CI: 0.512–0.56] reported lower odds of contraceptive continuation as compared to those with no births. Compared to women with no children living, those with 4 + children living had lower odds of contraceptive continuation [aOR: 0.62; CI: 0.57–0.67]. The study also found that the likelihood of contraceptive continuation was higher among those with secondary education [aOR: 1.08; CI: 1.04–1.12] as compared to those with no formal education. Contraceptive continuation was also higher among those who have information on choice [aOR: 3.91; CI: 3.82–4.01], and also higher among those who were undecided about having an additional child [aOR: 1.39; CI: 1.33–1.46]. Compared to West AfricaAngola, women from all other sub-regions were less likely to continue using contraceptives Comoros were more likely to continue with contraception [aOR: 1.49; CI: 1.24–1.78].

**Conclusion:**

To improve contraceptive continuation among women of reproductive age, countries in SSA must invest heavily in advocacy and dissemination of family planning messages, and information of choice. Also, much commitment should be directed towards enhancing the use of long-acting reversible contraceptive use.

## Background

The desire for large families is one of the major reasons for sub-Saharan Africa (SSA)’s high total fertility rates (TFR). Whereas most countries are hitting the later stage of the demographic transition, some countries in SSA are still struggling to get to this stage [1]. This has had an impact on their socio-economic development [2]. The TFR of SSA is estimated to be 4.7, with Niger and Cape Verde recording a TFR of 6.7 and 2.2, respectively [3].

Contraceptive usage has been a public health intervention for couples to have children by choice [4]. Potentially, it can reduce poverty and hunger in countries with high fertility [2]. This is why fertility regulation intervention received international recognition and donor support, especially in low-and middl-income countries [5]. Over the years, the increase in contraceptive method usage has resulted in a reduction in high-risk of unintended pregnancies, maternal and infant mortality, as well as improvement in the schooling and economic outcomes of girls and women especially [6].

Globally, out of the 1.9 billion reproductive-age women, 1.1 billion women have a family planning (FP) need, out of these women, 851 million use a modern contraceptive method and 85 million women use a traditional method [7]. Many studies have revealed that the unmet need for FP is a contributing factor to unwanted pregnancies among married women [8]. In 2020, the unmet need prevalence for FP in SSA was 24% [8]. That means twenty-four out of every 100 married women avoiding pregnancy at a particular period are not using any contraceptive method [9]. Indicator 3.7.1 of the Sustainable Development Goals women of reproductive age who have a FP need should be satisfied with modern methods) has increased from 74% in 2000 to 77% in 2020 universally [7]. Conversely, 33 million unplanned pregnancies are estimated to occur globally among women using any of the modern or traditional methods of contraception [10].

The unavailability of FP to women who need them sometimes results in discontinued use, often leading to unwanted pregnancies [11]. Also, some reasons attributed to FP method discontinuation includes: husband’s fear of infidelity or husband’s disapproval, “method-related concerns” like a woman’s fear of side effects and other health contraindications (e.g. fibroids) to a contraceptive method [12]. Myths and rumors (e.g., FP causes cancer and infertility) inhibit method continuation as well. Results from the 60 Demographic and Health Surveys (DHS) on contraceptive discontinuation purport a 38% discontinuation of reversible methods by month 12 and 64% by month 36 in 19 countries [13]. This phenomenon is defined as beginning and then discontinuing a contraceptive method while you are still at risk of unintended pregnancy [10]. According to the Population council, FP methods availability, enabling women switch method immediately, bringing the methods to women, and improving follow-up mechanisms are some interventions to increase FP continuation in our communities [14].

Previous findings on modern contraceptive discontinuation in Africa have concentrated on specific countries [15, 16] and method-specific contraceptives [10, 13]. This study aims to contribute to evidence on the determinants of contraceptive continuation among women in SSA using the recent available DHS data for sub-Saharan African countries. The selection of SSA for this analysis is based on its lower level of contraceptive uptake. The findings would highlight the need for enhanced donor support and stakeholder involvement, advocacy, and mobile FP services in the sub-Saharan African region.

## Methods

### Data source and study design

Data for the study were extracted from the most recent DHS of twenty-four countries in SSA. We pooled the data from the individual recode files in each of the 24 countries. The DHS is a comparatively nationally representative survey conducted in over 85 low-and-middle-income countries worldwide [17]. DHS employed a descriptive cross-sectional design. Respondents for the survey were recruited using a two-stage cluster sampling method. Detailed sampling technique has been highlighted in the literature [18]. Standardized structured questionnaires were used to collect data from the respondents on health indicators, including contraceptive use. We included a total of 128,362 women in this study (see Table 1). Only women with complete cases of variables of interest were included in the survey. The dataset used is freely available at https://dhsprogram.com/data/available-datasets.cfm. This manuscript was drafted with reference to the Strengthening the Reporting of Observational Studies in Epidemiology (STROBE) statement guidelines [19].

**Table 1:** Description of sample and prevalence of contraceptive continuation

Country	Survey year	Frequency	Percentage	Contraceptive continuation (%)
1. Angola	2015-16	1962	1.53	57.3
2. Burkina Faso	2010	3626	2.82	62.16
3. Benin	2018	4183	3.26	49.75
4. Burundi	2016-17	5975	4.65	42.88
5. Ethiopia	2016	5708	4.45	47.06
6. Ghana	2014	2912	2.27	59.55
7. Gambia	2013	3124	2.43	40.59
8. Guinea	2018	2785	2.17	36.55
9. Kenya	2014	8071	6.14	54.07
10. Comoros	2012	820	0.64	65.00
11. Liberia	2019-20	3828	2.98	51.83
12. Lesotho	2014	1959	1.53	60.44
13. Mali	2018	2640	2.06	50.19
14. Malawi	2015-16	16987	13.23	51.85
15. Nigeria	2018	8098	6.31	47.15
16. Niger	2012	3811	2.97	34.53
17. Namibia	2013	5239	4.08	63.79
18. Rwanda	2014-15	6890	5.37	49.96
19. Sierra Leone	2019	5118	3.99	60.84
20. Senegal	2010-11	2658	2.07	42.29
21. Tanzania	2015-16	6263	4.88	49.77
22. Uganda	2016	10103	7.87	42.38
23. Zambia	2018	8306	6.47	47.56
24. Zimbabwe	2015	7296	5.68	54.07
**Total**	**2010-20**	**128362**	**100.00**	**50.15**

### Variables

#### Outcome variables

The outcome variable used for this study was contraceptive continuation. This variable was measured as the percentage of women of reproductive age who continue to use the same method of contraception they have been using in the last 5 years prior to the survey within 12 months after starting to use it. In the DHS, this refers to users who continue using a contraceptive method within 12 months of beginning use during a specific episode of use which has been detailed in the DHS Statistics at https://dhsprogram.com/data/Guide-to-DHS-Statistics/index.htm#t=Contraceptive_Discontinuation.htm.

#### Explanatory variables

The explanatory variables used for this study were respondent’s age, marital status, educational level, place of residence, employment status, exposure to family planning messages, knowledge of family planning, information on choice of contraceptives, number of births in the last five years, number of living children, desire for more children, contraceptive methods used and sub-region. Place of residence and sub-region were contextual level variables and the remaining variables were individual level variables. The categories of each of the variables are shown in Table 2. The explanatory variables considered in this study were selected based on their association with contraceptive continuation from literature [20, 21] and also their availability in the DHS dataset.

### Statistical analysis

Data for the study were analysed using Stata version 16. First, a bar chart was used to show the prevalence of contraceptive continuation among reproductive aged women across the 24 countries. Next, the weighted frequencies and percentages for the explanatory variables were presented. Next, we presented the bivariate results on the distribution of contraceptive continuation among reproductive aged women across the explanatory variables using chi-square test of independence (Table 2). After this, we checked for multicollinearity among the explanatory variables using the variance inflation factor (VIF) and the results showed no evidence of high collinearity (Maximum VIF = 2.95, Minimum VIF = 1.06 and Mean VIF = 1.84). Finally, a two modelling binary logistic regression analysis was conducted to examine the association between the explanatory and outcome variables. The first model, Model I, was a multivariable binary logistic regression where only individual explanatory variables were used against the outcome variable to see the relationship between them. In the Model II, which is the complete model, was a multivariable binary logistic regression where all explanatory variables were used against the outcome variable to see the relationship between them (Table 3). The results were presented as adjusted odds ratio (aOR). All frequency distributions were weighted while the survey command (svy) in Stata was used to adjust for the complex sampling structure of the data in the regression analyses.

### Prevalence of contraceptive continuation among women in sub-Saharan Africa

Overall, the prevalence of contraceptive continuation among women in SSA was 50.2%. Comoros reported the highest prevalence of contraceptive continuation (65%) while Niger reported the lowest prevalence of continuation (34.5%) (see Table 1).

### Prevalence of contraceptive continuation among women in sub-Saharan Africa across explanatory variables

Table 2 shows the prevalence of contraceptive continuation among women in SSA across the explanatory variables. Except for exposure to family planning messages and knowledge on family planning, there were significant differences in the contraceptive continuation prevalence across all the explanatory variables. The prevalence was high among those aged 15–19 (62.5%), those not in union (57.1%), those with secondary education (53.4%), those in urban areas (52.1%), and those who are not working (51.0%). Also, the prevalence of contraceptive continuation was high among women who had information on choice (68.4%), those with zero birth in the last five years (59.8%), those who had none of their children alive (62.8%), those who were undecided about having more children (58.9%), and those who used LARCs (69.4%).

**Table 2:** Prevalence of contraceptive continuation among women in sub-Saharan Africa across explanatory variables

Variables	Weighted N	Weighted %	Contraceptive continuation	P-value
**Age (years)**				< 0.001
15–19	7771	6.1	62.5	
20–24	26,343	20.5	49.5	
25–29	32,876	25.6	45.5	
30–34	27,214	21.2	47.5	
35–39	19,701	15.3	51.6	
40–44	10,400	8.1	57.7	
45–49	4057	3.2	59.7	
**Marital status**				< 0.001
Not in union	25,684	20.1	57.1	
In union	102,678	79.9	48.4	
**Educational level**				< 0.001
No education	27,701	21.6	46.7	
Primary	51,012	39.7	49.2	
Secondary	41,312	32.2	53.4	
Higher	8337	6.5	51.2	
**Place of residence**				< 0.001
Urban	50,051	39.0	52.1	
Rural	78,311	61.0	48.9	
**Employment status**				0.001
Not working	31,859	24.8	51.0	
Working	96,503	75.2	49.9	
**Exposure to family planning messages**				0.264
Not exposed	60,350	47.0	50.3	
Exposed	68,012	53.0	50.0	
**Knowledge of family planning**				0.659
Poor	22	0.01	45.5	
Good	128,340	99.9	50.2	
**Information on choice**				< 0.001
No	75,443	58.8	37.3	
Yes	52,919	41.2	68.4	
**Births in the last 5 years**				< 0.001
None	28,361	22.1	59.8	
1	63,424	49.4	52.6	
2+	36,577	28.5	38.5	
**Number of living children**				< 0.001
None	10,180	7.9	62.8	
1	21,091	16.4	54.4	
2	27,072	21.1	46.3	
3	23,050	18.0	46.9	
4+	46,969	36.6	49.3	
**Desire for more children**				< 0.001
Wants within 2 years	16,806	13.1	44.5	
Wants after 2 + years	49,609	38.7	49.1	
Wants no more children	44,040	34.3	50.0	
Undecided	17,907	13.9	58.9	
**Method**				< 0.001
Long-Acting Reversible Contraception (LARC)	21,444	16.7	69.4	
Pills	22,106	17.2	40.9	
Injectables	54,297	42.3	45.3	
Other modern methods including condom	16,607	12.9	59.1	
Traditional method	13,908	10.8	43.6	
**Sub-Region**				< 0.001
West	44,510	34.7	50.7	
East	57,957	45.1	49.2	
Central	14,393	11.2	47.7	
South	11,502	9.0	56.2	

NB: Long-acting reversible contraceptive methods (LARCs) typically include intrauterine devices (IUDs) and implants, P-values are from chi-square test

### Predictors of contraceptive continuation in sub-Saharan Africa

Table 3 presents the results on the predictors of contraceptive continuation. Compared to adolescents, adult women aged 40–44 [aOR: 1.17; CI: 1.08–1.28] and 45–49 years [aOR: 1.24; CI: 1.13–1.37] had higher odds of contraceptive continuation. The odds of contraceptive continuation were lower among those working [aOR: 0.96; CI: 0.93–0.98] compared to those not working. Also, the study shows that the likelihood of contraceptive continuation was lower among those exposed to family planning messages compared to those not exposed [aOR: 0.91; CI: 0.88–0.93].

**Table 3:** Logistic regression on the predictors of contraceptive continuation in sub-Saharan Africa

Variables	Model I	Model II
**Age (years)**		
15–19	Reference (1.0)	Reference (1.0)
20–24	0.59^***^ (0.56–0.62)	0.81^***^ (0.76–0.89)
25–29	0.50^***^ (0.48–0.53)	0.78^***^ (0.73–0.83)
30–34	0.54^***^ (0.52–0.57)	0.85^***^ (0.79–0.91)
35–39	0.64^***^ (0.61–0.68)	0.99 (0.92–1.06)
40–44	0.82^***^ (0.77–0.87)	1.17^***^ (1.08–1.28)
45–49	0.89^**^ (0.82–0.96)	1.24^***^ (1.13–1.37)
**Marital status**		
Not in union	Reference (1.0)	Reference (1.0)
In union	0.70^***^ (0.69–0.72)	0.98 (0.95–1.02)
**Educational level**		
No education	Reference (1.0)	Reference (1.0)
Primary	1.10^***^ (1.07–1.14)	1.03 (1.00-1.07)
Secondary	1.31^***^ (1.27–1.35)	1.08^***^ (1.04–1.12)
Higher	1.20^***^ (1.14–1.26)	0.91^***^ (0.86–0.97)
**Employment status**		
Not working	Reference (1.0)	Reference (1.0)
Working	0.96^***^ (0.93–0.98)	0.96^***^ (0.93–0.98)
**Exposure to family planning messages**		
Not exposed	Reference (1.0)	Reference (1.0)
Exposed	0.99 (0.97–1.01)	0.91^***^ (0.88–0.93)
**Knowledge of family planning**		
Poor	Reference (1.0)	Reference (1.0)
Good	1.21 (0.52–2.80)	0.79 (0.32–1.93)
**Information on choice**		
No	Reference (1.0)	Reference (1.0)
Yes	3.64^***^ (3.55–3.73)	3.91^***^ (3.82–4.01)
**Births in the last 5 years**		
None	Reference (1.0)	Reference (1.0)
1	0.75^***^ (0.72–0.77)	0.86^***^ (0.83–0.90)
2+	0.42^***^ (0.41–0.43)	0.54^***^ (0.51–0.56)
**Number of living children**		
None	Reference (1.0)	Reference (1.0)
1	0.71^***^ (0.67–0.74)	0.84^***^ (0.79–0.89)
2	0.51^***^ (0.49–0.54)	0.67^***^ (0.63–0.72)
3	0.52^***^ (0.50–0.55)	0.65^***^ (0.6–0.70)
4+	0.58^***^ (0.55–0.60)	0.62^***^ (0.57–0.67)
**Desire for more children**		
Wants within 2 years	Reference (1.0)	Reference (1.0)
Wants after 2 + years	1.20^***^ (1.16–1.24)	1.21^***^ (1.16–1.25)
Wants no more children	1.24^***^ (1.20–1.29)	1.15^***^ (1.10–1.20)
Undecided	1.78^***^ (1.71–1.86)	1.39^***^ (1.33–1.46)
**Contraceptive method**		
Long-Acting Reversible Contraception (LARC)	Reference (1.0)	Reference (1.0)
Pills	0.31^***^ (0.29–0.32)	0.34^***^ (0.33–0.36)
Injectables	0.37^***^ (0.35–0.79)	0.42^***^ (0.40–0.43)
Other modern methods including condom	0.64^***^ (0.61–0.66)	0.72^***^ (0.68–0.75)
Traditional method	0.34^***^ (0.33–0.36)	0.50^***^ (0.47–0.52)
**Place of residence**		
Urban		Reference (1.0)
Rural		0.99 (0.97–1.02)
**Sub-region**		
West Africa		0.78^***^ (0.74–0.82)
East Africa		0.69^***^ (0.66-0.72)
Central Africa		0.88^***^ (0.84–0.93)
Southern Africa		Reference (1.0)

* p < 0.05, ** p < 0.01, *** p < 0.001

Compared to women who used LARCs, women who used pills [aOR: 0.34; CI: 0.33–0.36], injectable [aOR: 0.42; CI: 0.40–0.43], other modern contraceptives [aOR: 0.72; CI: 0.68–0.75] or traditional methods [aOR: 0.50; CI: 0.47–0.52] were less likely to continue with their contraception. Women with one birth [aOR: 0.86; CI: 0.83–0.90] and those with 2 + births in the last five years [aOR: 0.54; CI: 0.51–0.56] reported lower odds of contraceptive continuation as compared to those with no births. Compared to women with no children living, those with 4 + children living had lower odds of contraceptive continuation [aOR: 0.62; CI: 0.57–0.67]. The study also found that the likelihood of contraceptive continuation was higher among those with secondary education [aOR: 1.08; CI: 1.04–1.12] as compared to those with no formal education. It was also higher among those who have information on choice [aOR: 3.91; CI: 3.82–4.01], and also higher among those who were undecided about having an additional child [aOR: 1.39; CI: 1.33–1.46]. Compared to West Africa, women from all other sub-regions were less likely to continue using contraceptives.

## Discussion

The present study examined the determinants of contraceptive continuation among women in SSA. Overall, the prevalence of contraceptive continuation was 50.2%. The estimated prevalence of contraceptive continuation in this study is moderately low when compared to estimated prevalence in some SSA countries such as the Democratic Republic of Congo (86.1%) [22]. Similarly, our estimated prevalence of contraceptive continuation is lower than the 59% that was reported in a related study [23]. This moderately low prevalence of contraceptive continuation is explained by the associated factors that emerged significant in our analyses.

Type of contraceptive was a significant factor associated with contraceptive continuation. Using short term contraceptives (i.e., pills and injectable) or traditional methods was associated with lower odds of continuation as compared to those used LARCs. The result corroborates previous studies conducted in Kenya [24] and Papua New Guinea [25]. Short term and traditional contraceptives have lower efficacy rates. For instance, evidence shows that implants are 120 times more effective than injectables, and 180 times more effective than pills [26, 27]. Hence, it is possible that women who patronise these forms of contraceptives will switch to other methods which have higher efficacy rates [24]. Thus, explaining why the likelihood contraceptive continuation was lower among women who used short term and traditional methods of contraception. Another possible explanation could be that, short term methods require much commitment and a conscious effort to maintain consistency [28]. Therefore, women who use short term methods are prone to forget or skip its use, thereby resulting in lower likelihood of continuation.

Our study revealed that there was statistically significant association between desire for more children and contraceptive continuation. Women who were undecided about having an additional child were 1.35 more likely to continue with one method of contraception as compared to their counterparts who desired another child within 2 years. Our result is corroborated by a related study in the Democratic Republic of Congo [22]. This finding is unsurprising because, the purpose of contraceptives is to prevent pregnancy. Therefore, women who desire another child in the shortest time period would want to discontinue contraceptive use in order to boost their chances of getting pregnant.

Consistent with previous studies [25, 29], we found higher odds of contraceptive continuation among women who had information on choice, compared to those who did not have access to information on choice. A plausible explanation for this finding could be that, having access to information on choice exposes women to all the contraceptives available, their benefits, and risks. Hence, women are able to make an informed choice about the best contraceptive that they can use without discontinuing. Relatedly, we found statistically significant association between exposure to family planning messages and contraceptive continuation, with those exposed to such messages having lower odds of continuation. Similar findings have been reported in Indonesia [30].

Older women in the reproductive age (40–49 years) were more likely to have higher contraceptive continuation as compared to adolescent girls (15–19 years). This finding is synonymous to a related study conducted in Ethiopia [31]. Adolescents often have limited access to contraceptives as compared to older adults, and thus, explain the high likelihood of contraceptive continuation among those aged 40–49 years. Our study as revealed that having secondary education was associated with higher contraceptive continuation. The result is analogous to earlier findings from Ethiopia [32]. A plausible explanation could be that, higher formal education provides knowledge to women, improves access to resources that may be utilised to get contraception, and boosts their sexual negotiation power and involvement in decision-making processes [33].

Lower odds of contraceptive continuation were reported among women with one birth and 2 + births within the last five years. This is consistent with a related study conducted in Ghana [25] and Ethiopia [34]. Similar to this finding, our study revealed that women with four or more children living were less likely to continue with contraceptives.

### Policy implications

The result from our study that information of choice is associated with contraceptive continuation underscores the need for countries in SSA to strengthen family planning and contraceptive use advocacy as that would empower women and arm them with accurate information necessary to make an informed decision. Since the use of LARCs was associated with higher likelihood of contraceptive continuation, policies and interventions must be focused to improve access to LARCs as well as demystifying women of misconceptions about LARCs. Additionally, there is a need for policy makers and implementers to take into account how the side effects of contraceptives influence the its continuous use among women. The study also highlights the need to improve access to contraceptives in rural areas.

### Strengths and limitations

Because of the cross-sectional study methodology used, causal conclusions cannot be drawn from this study. Furthermore, because the study is retrospective, there is the possibility of recollection bias. Nonetheless, this study provides nationally representative coverage of contraceptive cessation among women of reproductive age in SSA.

## Conclusion

Our study reports 50.2% contraceptive continuation among women in SSA. The factors associated with contraceptive continuation include age, rural residency, working, having secondary education, exposure to family planning messages, having information on choice, desire for more children and then type of contraceptive used. To improve contraceptive continuation among women of reproductive age, countries in SSA must invest heavily in advocacy and dissemination of family planning messages, and information of choice. Also, more emphasis should be directed towards enhancing LARCs use.

## Data Availability

The dataset used is free available at https://dhsprogram.com/data/available-datasets.cfm.
